# The Impact of Small Spinal Curves in Adolescents Who Have Not Presented to Secondary Care: A Population-Based Cohort Study

**DOI:** 10.1097/BRS.0000000000001330

**Published:** 2016-05-10

**Authors:** Emma M. Clark, Jon H. Tobias, Jeremy Fairbank

**Affiliations:** ∗ Musculoskeletal Research Unit, School of Clinical Science, University of Bristol, Southmead Hospital, Bristol, UK; † Nuffield Department of Orthopaedics, Rheumatology and Musculoskeletal Sciences, University of Oxford, Nuffield Orthopaedic Centre, Oxford, UK.

**Keywords:** ALSPAC, cohort, days of school, DSM, DXA, epidemiology, impact, pain, scoliosis, small curves

## Abstract

**Study Design.:**

A prospective, population-based, birth cohort study.

**Objective.:**

The aim of this study was to identify whether there is any hidden burden of disease associated with smaller spinal curves.

**Summary of Background Data.:**

Adolescent idiopathic scoliosis is present in 3% to 5% of the general population. Large curves are associated with increased pain and reduced quality of life. However, no information is available on the impact of smaller curves, many of which do not reach secondary care.

**Methods.:**

The Avon Longitudinal Study of Parents and Children (ALSPAC) recruited over 14,000 pregnant women from the Bristol area of South-West England between 1991 and 1992 and has followed up their offspring regularly. At age 15, presence or absence of spinal curvature ≥6 degrees in the offspring was identified using the validated dual-energy X-ray absorptiometry Scoliosis Measure on 5299 participants. At age 18, a structured pain questionnaire was administered to 4083 participants. Logistic regression was used to investigate any association between presence of a spinal curve at age 15 and self-reported outcomes at age 18 years.

**Results.:**

Full data were available for 3184 participants. Two hundred two (6.3%) had a spinal curve ≥6 degrees and 125 (3.9%) had a curve ≥10 degrees (median curve size of 11 degrees). About 46.3% reported aches and pains that lasted for a day or longer in the previous month. About 16.3% reported back pain. Those with spinal curves were 42% more likely to report back pain than those without (odds ratio 1.42, 95% confidence interval 1.00–2.02, *P* = 0.047). Those with spinal curves had more days off school and were more likely to avoid activities that caused their pain.

**Conclusion.:**

Our results highlight that small scoliotic curves may be less benign than previously thought. Teenagers with small curves may not present to secondary care, but are nonetheless reporting increased pain, more days off school, and avoidance of activities. These data suggest that we should reconsider current scoliosis screening and treatment practices.

**Level of Evidence:** 2

Idiopathic scoliosis is classified according to age of onset,^1^ and adolescent idiopathic scoliosis (AIS), with an onset between age 10 years and skeletal maturity, is the most common type. It is well recognized that although mortality rates for individuals with AIS are comparable to that of the general population,^2^ scoliosis is not always a benign structural abnormality. For example, the presence of a large scoliotic curve (mean 50 degrees) is associated with a reduction in both static and dynamic pulmonary function measures.^3^ The majority of people with large AIS curves seem to develop back pain as an adult,^4,5^ although this may only be mild or moderate.^6^ However, back pain associated with AIS has also been shown to be associated with increased requirement for physiotherapy, disability pensions, and unemployment.^7^ Furthermore, adults with AIS can experience a range of psychosocial impacts,^8^ including an increase in depressive symptoms^9^ and body image disturbance.^10^

However, there are methodological issues with these studies. Many do not have a control group to compare against,^3,7^ and of those that do, the study design used is case control wherein selection of appropriate controls that are otherwise representative of the general population may be problematic.^6,9,10^ Two systematic reviews of studies looking at health-related quality of life and psychosocial outcomes in people with scoliosis identified 57^8^ studies ranging in size from 16 to 685 participants, and 15^11^ patient cohorts (case series). In all studies (case series or case control), the cases were identified *via* secondary care spinal units. It is likely that people who present to spinal units either have symptoms or were identified by school-based screening by clinical examination using the Adam's forward bending test followed by scoliometer measurement of the angle of trunk inclination (ATI). However, a 10-year follow-up examination of children identified with possible scoliosis using these clinical tests found that the negative predictive value was poor.^12^ This suggests that even in the studies based on people identified with AIS through school-based screening programs, people with spinal curves are likely to have been missed, and these missing people are likely to be those with fewer symptoms and smaller curves. It is therefore possible that we are overestimating the impact of scoliosis in the general population.

Population-based studies of scoliosis are difficult because the gold-standard diagnostic test of standing radiographs cannot be performed on entire populations because of the relatively high exposure to ionizing radiation.^13^ However, we have developed a method for measuring spinal curvature using total-body dual-energy X-ray absorptiometry (DXA) supine scans for research purposes.^14^ This now allows us to assess the impact of small spinal curves in general populations, irrespective of whether they have presented to spinal units. The aim of this present study was to assess the prospective association between spinal curvature at age 15 and pain at age 18 utilizing a large birth cohort, to identify whether there is any hidden burden of disease, or alternatively to identify whether small spinal curves are of little or no consequence in the general population.

## METHODS

### Study Population

The Avon Longitudinal Study of Parents and Children (ALSPAC) is a geographically based UK cohort that recruited pregnant women residing in Avon (South-West England) with an expected date of delivery between April 1991 and December 1992.^15^ A total of 14,541 pregnancies were enrolled with 14,062 children born (see www.alspac.bris.ac.uk for more information). The study website contains details of all the data that are available through a fully searchable data dictionary (http://www.bris.ac.uk/alspac/researchers/data-access/data-dictionary/). Ethical approval for the study was obtained from the ALSPAC Ethics and Law Committee and the Local Research Ethics Committees.

### Exposure Measure: Spinal Curvature Using the DXA Scoliosis Method (DSM)

As previously described,^14^ all total-body DXA scans from the “Aged 15” Research Clinic (carried out in a standard supine manner by trained technicians) were triaged into likely scoliosis or not, by visual evaluation to distinguish true curves from positioning errors. Scans triaged as likely scoliosis had an angle size measured using a modified-Ferguson method,^16^ as DXA images are low resolution and individual end plates cannot be identified and so the standard Cobb method cannot be used. To perform the modified Ferguson method, first a “normal spine line” (NSL) was drawn through the center of the spine level with the first rib attachment, down to the center of the spine at L5. Next, the apex of the curve was identified as the center of the spinal column most translated away from the NSL. Lines were then drawn from the apex of the curve to the NSL at the point where the center of the spinal column first touched the NSL on return from the apex. In double or triple curves, the spine did not always return to the NSL before the next curve started, so the center of the spinal column at the judged point of inflection was used as the end of the curvature. Also, as previously published,^14^ precision was assessed on 174 children who had repeat DXA scans taken 2 to 6 weeks apart, and substantial agreement in identifying those with scoliosis was seen (kappa 0.74). Of repeat angle measures, 95% were within 5 degrees. Comparison with the gold-standard of standing spinal radiographs showed that this DXA-based method underestimates curve size, with an approximate 30% reduction due to supine position, and an additional 10% from use of the modified-Ferguson, suggesting that a cut-off of 6 degrees is equivalent to the conventional criterion of 10 degrees. For this paper, a spinal curvature of ≥6 degrees was used as our primary exposure, and sensitivity analyses were carried out using a cut-off of ≥10 degrees. Data were also collected on direction of convexity and site of curve.

### Primary Outcome Measure: Pain

As previously described,^17^ a structured pain questionnaire assembled from domains and scales taken from questionnaires previously validated in UK populations was administered to participants at the “Aged 18” ALSPAC Research Clinic. Participants were asked whether they had any aches or pains that lasted a day or longer in the previous month. If the answer was yes, then they were then asked to indicate the site of pain on a diagram. Back pain was defined as a mark anywhere on the diagram over the upper or lower midline spine area. The upper back was defined as anywhere marked from above the waist to below the shoulders. The lower back was defined as below the waist to the top of the legs, including the buttocks. Other discrete areas of pain identified included shoulder, buttock, and neck. Participants were asked to indicate the intensity of their worst pain over the last 6 months using a visual analog scale of 0 (no pain) to 10 (pain as bad as could be).

### Secondary Outcome Measures

Days off activities at age 18 as a result of pain were identified by asking participants how many days they had been kept from usual activities (school, work, or housework). Impact was identified by asking whether troublesome pain has resulted in avoidance of activities, worry that something harmful is happening, and fear of moving due to pain.

### Other Measures

Ethnic group was categorized as white or nonwhite. Gender was obtained from birth notifications. Mother's highest educational qualifications was also assessed at 32 weeks of gestation and was coded on a 5-point ascending scale on which levels 1, 2, and 3 referred to educational qualifications generally gained at school by 16 years of age, level 4 to qualifications gained at school at 18 years of age, and level 5 to university degrees. Age was calculated from date of birth. The short Moods and Feelings Questionnaire was analyzed in the standard manner to generate a validated indication of depression at age 16.^18^

### Statistical Analysis

All statistical analyses were performed using Stata 11.2 (StataCorp, College Station, TX). Chi-squared tests were used for simple associations between categorical exposure and outcome. Odds of exposure to spinal curvature at age 15 in those with and without pain at age 18 were calculated. Logistic regression was used to calculate odds ratios (ORs) and 95% confidence intervals (CIs) to describe the association between spinal curvature and presence or absence of back pain. Multivariable logistic regression was used to identify independent associations.

#### Sensitivity analyses

All analyses using spinal curvature as a binary variable were rerun using a stricter definition of spinal curvature as ≥10 degrees. For some analyses, spinal curvature was used as a continuous variable. In addition, all analyses were rerun after excluding those children who were told at the “Aged 13” and “Aged 18” Research Clinics that their Adams forward bending test was not normal (ATI ≥7 degrees, n = 33), as these participants may have been aware that they had scoliosis that could have introduced bias.

## RESULTS

### General Description of the Cohort

Full data were available on 3184 participants: 56.8% were female and 4.2% nonwhite, which reflects the local population; and 49.4% of mothers had qualifications gained at school at 18 years of age or university degrees (see Table 1). As expected, more females than males had spinal curvature (ratio of 2.3 : 1, *P* < 0.001). Just under half the cohort reported aches or pains that lasted a day or longer in the past month. Also as expected, more females than males had indicators of depression (*P* < 0.001).

### Description of Spinal Curves in the Cohort

**Figure 1:**
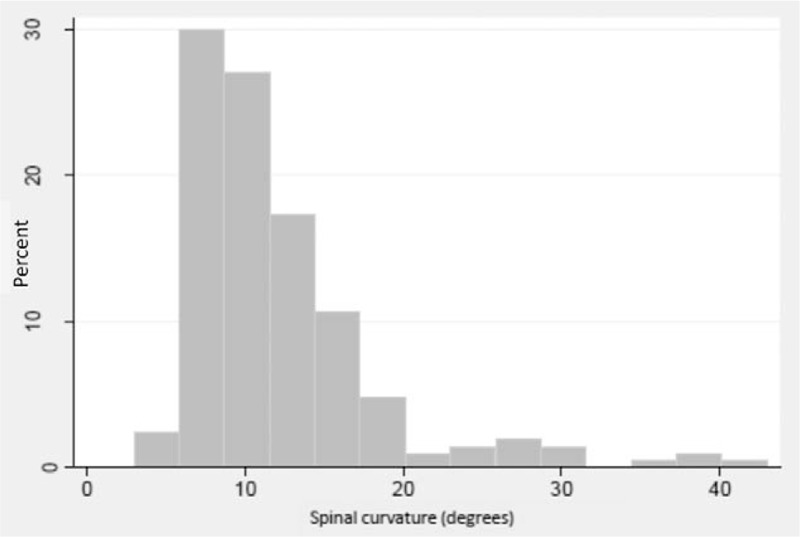
Distribution of size of spinal curve (degrees) as measured by a modified Fergusson technique on supine total-body DXA images in this population of adolescents.

At age 15, 202 of 3184 (6.3%) had a spinal curvature of ≥6 degrees and 125 (3.9%) had a curve of ≥10 degrees. The median curve size was 11 degrees with an interquartile range of 8 to 14 degrees (see Figure 1), and there were 11 participants with a curve ≥25 degrees. As previously described,^14^ 140 of 202 (69.3%) had single curves, and of these, 57.4% were to the right and 41.4% were thoracic. As previously reported,^19^ there was no association between ethnicity, maternal education or pubertal stage, and spinal curvature. However, also as previously described, body weight was inversely associated with scoliosis, due to a combination of lower fat mass and lower lean mass.^19^ No association was seen between presence of scoliosis at 15 and poorer mental health at age 16: 12.1% of those without scoliosis had indications of depression compared with 13.7% of those with scoliosis (*P* = 0.545).

### Description of Pain and Other Impact in the Cohort

At age 18, 519 of 3184 (16.3%) reported back pain lasting for a day or longer in the past month. About 35.9% rated their pain as very or extremely troublesome, with the worst pain being rated as ≥5 out of 10 by 83.5%. Those with back pain had a slightly higher body mass index (BMI), but no association was seen with gender, ethnicity, or socioeconomic status (see Table 2). In addition, 448 (14.1%) reported shoulder pain, 227 (8.7%) buttock pain, and 170 (5.3%) neck pain. Of those who reported pain, 85.8% reported 0 to 6 days off, 8.3% 7 to 14 days, 3.0% 15 to 30 days, and 2.9% >30 days off. Those with 7 or more days off because of pain were more likely to be female and more likely to be of nonwhite ethnicity (see Table 2).

### Association Between Spinal Curves and Pain

**Figure 2:**
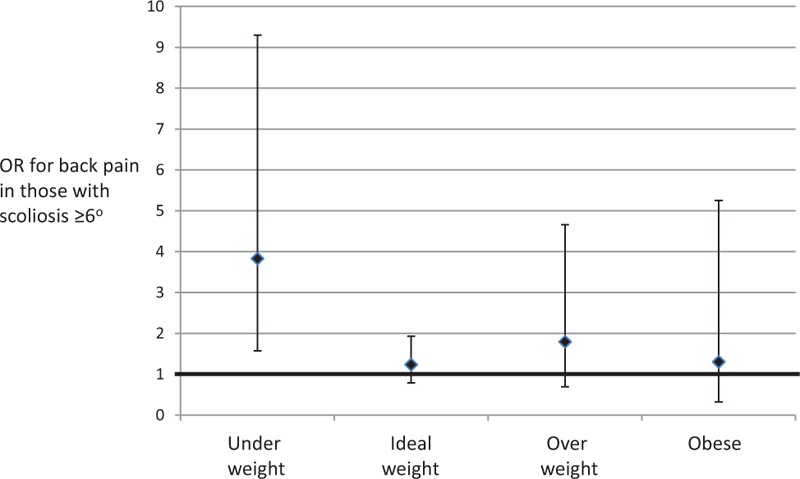
Odds ratios (ORs) for association between scoliosis ≥6 degrees at age 15 and back pain at age 18 stratified by standard BMI categories into those who are underweight, ideal weight, overweight, and obese. Error bars are 95% confidence intervals, and the null value of 1.0 is illustrated by the bold horizontal line.

Those with spinal curves at age 15 were 42% more likely to report back pain at age 18. Adjustment for age, gender, and ethnicity did not alter the strength of association (see Table 3). Stratification by BMI showed the association between spinal curve and back pain was mainly driven by those who were underweight (see Figure 2), with a nearly 3-fold increase in back pain in those with low body weight and spinal curve (OR 3.82, 95% CI 1.57–9.30, *P* = 0.003). Those with lumbar curves were slightly more likely to report lower back pain, and those with thoracic curves were slightly more likely to report upper back pain, but this did not reach statistical significance. No association was seen between spinal curve and shoulder, buttock, or neck pain. Rerunning the analysis excluding those who may have known they had a curve or using a higher spinal curve cut-off of ≥10 degrees did not alter the results. Rerunning the analysis after excluding larger curves still showed an association between small spinal curves (6–10 degrees) and back pain (OR 1.65, 95% CI 1.04–2.61, *P* = 0.034). Further adjustment for depression did not change the results.

A positive association was seen between increasing size of spinal curve and increasing intensity of worst pain experienced, after adjusting for age and BMI (*P* = 0.036): curves of <6 degrees had an intensity of 6.5, curves of 6 to 10 degrees had an intensity of 6.7, curves of 10–25 degrees had an intensity of 7.1, and curves of >25 degrees had an intensity of 8.3.

### Other Impact of Spinal Curves

Those with spinal curves at age 15 were twice as likely to have days off school, work, or housework at age 18. Results were barely changed by adjustment for age, gender, ethnicity, and BMI (see Table 3). Those with spinal curves were also more likely to avoid activities because of their pain, and more likely to think something harmful was happening to them. Rerunning the analysis excluding those who may have known they had a curve or using a higher spinal curve cut-off of ≥10 degrees did not alter the results.

## DISCUSSION

We present the results of the first population-based study of the impact of small spinal curves and identify a hidden burden of disease: small spinal curves that may not present to secondary care are nonetheless associated with increased pain, more days off school and avoidance of activities. Our results are important because school attendance predicts academic success in terms of school grades,^20^ and this may in turn influence early career development and thus have far-reaching economic impacts for both the individual concerned and society as a whole.

Our results agree with previous literature that has identified an association between AIS and back pain during adolescence^21^ and adulthood,^6,21,22^ but we extend knowledge by providing the first population-based data. Previous studies have either been cross-sectional or case control. The case–control studies are likely to have bias due to recruitment of cases from spinal surgery units and *via* recruitment of controls. The large cross-sectional study of 43,000 school pupils in Japan^21^ is likely to have bias through its identification of people with scoliosis *via* school-based screening, as the prevalence of scoliosis in this study was 0.16%, much lower than expected. Our study adds to this by highlighting the importance of small curves. The mean curve size in our sample (11 degrees, roughly equivalent to 15 degrees measured by the Cobb method) is much smaller than that seen in previous studies.

Back pain is a major public health problem: the 2013 Global Burden of Disease Study reported back pain as a leading cause for Years Lived with Disability (YLDs) in every one of the 188 countries studied,^23^ and back pain causes more disability globally than any other condition.^24^ For the most part, the causes are unknown. These data suggest that small spinal curves are related to back pain and suggest that there is a case to screen and then investigate the early treatment of these curves to see whether impact can be reduced. However, this raises 2 issues that are unresolved: how to screen effectively for small spinal curves and how to treat them once identified. There is no consensus on the best way to screen. School-based screening programs are undertaken in some countries using measurement of the ATI in the forward bending position or in the sitting position. However, a 10-year follow-up evaluation of such children found that the negative predictive value was poor and concluded that the ATI should not be used as diagnostic criteria for detection of scoliosis because of the unacceptable number of false-negatives.^12^ The gold standard of standing radiographs cannot be performed on entire populations because of the exposure to relatively high levels of ionizing radiation,^13^ although a lower radiation standing imaging technique has been developed (the EOS 2D/3D system, http://www.eos-imaging.com) but is not currently recommended for routine use in many countries. An alternative method is surface topography^25^ (the use of light and shadows on the back to produce a 3-D description of back shape), but this requires specialist equipment and interpretation. DXA capacity would need to increase for our supine DXA method to be useful for national screening programs. If small curves are important in relation to adolescent back pain, then better methods of screening are needed.

In addition, screening should only be implemented if there is a treatment that works and is cost-effective. The current nonoperative techniques of bracing and physiotherapy are the most likely candidates, but more evidence is needed for the effectiveness in people with small curves. Bracing^26^ is generally used in people with curves >25 degrees and our study had only 11 participants with curves of this size. Physiotherapy and exercise therapy may also be helpful, although again there is little evidence to support this in people with small curves. However, it may be that a global exercise treatment or other intervention is the best way of reducing adolescent back pain and that identification of small curves is not necessary to prevent or reduce the incidence of adolescent back pain. Alternatively, if these small curves in adolescence contribute to the later appearance of adult scoliosis, then there may be an argument for identification of small curves by DXA or equivalent methods in adolescents to reduce the risk of adult back pain consequent on adult spinal deformity. Clearly, much more work is needed to address these questions. The validated scoliosis outcome measure Scoliosis Research Society 22 (SRS-22)^27^ includes questions about pain and level of activity. Our results suggest that the use of SRS-22 as an outcome in all future intervention studies for people with small spinal curves should be mandatory.

There are limitations to this study. As images from the DXA machine are taken in the supine position, it is possible that identified curves are postural due to back pain, and as we did not ask about pain at age 15, we are unable to exclude reverse causality. However, it is likely that pain-related curves will correct in the supine position. A further limitation of our study includes loss of a large proportion of the original ALSPAC cohort, which may have introduced bias, for example, by a preferential dropout of children from families of lower socioeconomic status. In common with all observational studies, we cannot exclude confounding and chance.

In conclusion, we present the first results from a population-based study of the impact of small spinal curves and identify an important hidden burden of disease. Our results highlight that small scoliotic curves that may not present to secondary care are nonetheless associated with increased pain, more days off school, and avoidance of activities. This study generates far-ranging questions on the value of school-based screening on public health grounds, the need for further research into nonsurgical interventions designed to reduce pain and increase participation in adolescents with small spinal curves, and the importance of patient-related outcome measures in all future studies.

**Key Points**

- Spinal curvature can be measured from total-body dual-energy X-ray absorptiometry (DXA) scans for research purposes.
- Small spinal curves at age 15 are associated with back pain at age 18 in the general population.
- Spinal curvature at age 15 is also associated with days off school and avoidance of activities at age 18.

## Figures and Tables

**TABLE 1:** General Descriptives of the Study Population Divided Into Males and Females

	Males n = 1377	Females n = 1807	Entire Cohort n = 3184
	Mean (SD)	Mean (SD)	Mean (SD)
	15.5 (0.3)	15.5 (0.3)	15.5 (0.3)
Age at Time of Spinal Curvature Measure (yrs)	N (%)	N (%)	N (%)
Ethnicity			
White	1218 (96.2)	1586 (95.5)	2804 (95.8)
Nonwhite	48 (3.8)	74 (4.5)	122 (4.2)
Maternal education			
1	132 (10.3)	166 (9.9)	298 (10.1)
2	86 (6.7)	116 (6.9)	202 (6.8)
3	396 (30.9)	602 (35.8)	998 (33.7)
4	381 (29.7)	469 (27.9)	850 (28.7)
5	287 (22.4)	327 (19.5)	614 (20.7)
Spinal curve ≥6^o^			
No	1327 (96.4)	1655 (91.6)	2982 (93.7)
Yes	50 (3.6)	152 (8.4)	202 (6.3)
Pain lasting a day or longer in the last month			
No	790 (57.4)	920 (50.9)	1710 (53.7)
Yes	587 (42.6)	887 (49.1)	1474 (46.3)
Mental health			
Fine	957 (93.3)	1247 (84.1)	2204 (87.8)
Indications of depression	69 (6.7)	236 (15.9)	305 (12.2)

SD indicates standard deviation.

**TABLE 2:** Description of Those With and Without Back Pain and Those With and Without Days Off School in the Last 6 Months Because of Pain

	No Back Pain n = 2665	Back Pain n = 519	*P* for Difference	0–6 days off school n = 1340	7 or more days off school n = 198	*P* value for difference
	Mean (SD)	Mean (SD)	Mean (SD)	Mean (SD)	Mean (SD)	Mean (SD)
	17.7 (0.4)	17.8 (0.4)	P = 0.074	17.7 (0.4)	17.8 (0.4)	*P* = 0.079
Age at Time of Back Pain Measure (yrs)	N (%)	N (%)	N (%)	N (%)	N (%)	N (%)
**Gender**			*P* = 0.091			*P* < 0.001
Male	1170 (43.9)	207 (39.9)		554 (41.3)	56 (28.3)	
Female	1495 (56.1)	312 (60.1)		789 (58.7)	142 (71.7)	
**Maternal education**			*P* = 0.241			*P* = 0.125
1	239 (9.6)	59 (12.5)		131 (10.6)	23 (12.2)	
2	167 (6.7)	35 (7.4)		70 (5.7)	16 (8.5)	
3	836 (33.6)	162 (34.4)		413 (33.4)	72 (38.3)	
4	723 (29.0)	127 (27.0)		366 (19.6)	49 (26.1)	
5	526 (21.1)	88 (18.7)		256 (20.7)	28 (14.9)	
**Ethnicity**			P = 0.258			P = 0.043
White	2360 (96.0)	444 (94.9)		1178 (96.2)	171 (92.9)	
Nonwhite	98 (4.0)	24 (5.1)		47 (3.8)	13 (7.1)	
**BMI at time of back pain measure**			*P* = 0.005			*P* = 0.455
Underweight (<18.5)	237 (9.1)	36 (7.1)		98 (7.5)	11 (5.8)	
Ideal weight (18.5–24.9)	1837 (70.4)	350 (68.6)		912 (69.4)	136 (71.6)	
Overweight (25.0–29.9)	388 (14.9)	75 (14.7)		202 (15.4)	24 (12.6)	
Obese (≥30)	147 (5.6)	49 (9.6)		102 (7.8)	19 (10.0)	

**TABLE 3:** Association Between Spinal Curve at Age 15 and Back Pain, Days Off School, and Other Impacts at Age 18

	No Spinal Curve (n = 2982)	Spinal Curve (n = 202)	*P*	(A) Unadjusted	(B) Adjusted for Age, Gender, Ethnicity	(C) Additionally Adjusted for BMI
	N (%)	N (%)		OR (95% CI), *P*	OR (95% CI), *P*	OR (95% CI), *P*
Outcomes at age 18						
**Back pain**			*P* = 0.047	1.42 (1.00–2.02), *P* = 0.048	1.47 (1.01–2.15), *P* = 0.043	1.56 (1.07–2.28), *P* = 0.022
Yes	476 (16.0)	43 (21.3)				
No	2506 (84.0)	159 (78.7)				
**Days off**			P = 0.009	1.98 (1.18–3.33), *P* = 0.010	1.79 (1.03–3.11), *P* = 0.039	1.92 (1.10–3.34), *P* = 0.022
0–6	1268 (87.7)	72 (78.3)				
7 or more	178 (12.3)	20 (21.7)				
**Avoidance of activities**			*P* = 0.006	1.60 (1.14–2.24), *P* = 0.006	1.60 (1.12–2.30), *P* = 0.010	1.64 (1.14–2.37), *P* = 0.007
Sometimes, often or always	499 (17.1)	49 (24.8)				
Never of hardly ever	2427 (82.9)	149 (75.3)				
**Afraid of their pain**			*P* = 0.196	1.28 (0.88–1.86), *P* = 0.197	1.22 (0.82–1.82), *P* = 0.329	1.22 (0.82–1.84), *P* = 0.329
Sometimes, often, or always	437 (15.0)	36 (18.4)				
Never of hardly ever	2487 (85.1)	160 (81.6)				
**Think something harmful is happening**			*P* = 0.044	1.45 (1.01–2.09), *P* = 0.045	1.62 (1.10–2.39), *P* = 0.014	1.62 (1.09–2.40), *P* = 0.016
Sometimes, often, or always	428 (14.7)	39 (20.0)				
Never of hardly ever	2487 (85.3)	156 (80.0)				

Results are number and percentage with *P* value for difference calculated by Chi-square. Also shown are odds ratios (ORs) for outcomes at age 18 (A) unadjusted, (B) adjusted for age, gender, and ethnicity, and (C) adjusted for age, gender, ethnicity, and BMI. 95% CI indicates 95% confidence interval; OR, odds ratio.
